# Event-Related Potentials in COA’s

**Published:** 1997

**Authors:** Bernice Porjesz, Henri Begleiter

**Affiliations:** Bernice Porjesz, Ph.D., is an assistant professor in the Department of Psychiatry, State University of New York Health Science Center, Brooklyn, New York. Henri Begleiter, M.D., Ph.D., is a professor of psychiatry and neuroscience in the Department of Psychiatry, State University of New York Health Science Center, Brooklyn, New York

**Keywords:** children of alcoholics, evoked potential, brainwaves, hereditary factors, genetic markers, AOD use susceptibility, Cloninger’s typology, risk assessment, nervous system, literature review

## Abstract

Evidence suggests that people at risk for developing alcoholism can be distinguished from those not at risk by measuring the electrical activity of the brain. Event-related potentials (ERP’s) are brain electrical signals produced in response to specific sensory stimuli. Reduced voltage of an ERP called P300, or P3, appears to characterize offspring of alcoholic families, regardless of whether the offspring are themselves alcoholic. Reduced P3 may indicate susceptibility to alcoholism and may elucidate mechanisms of alcohol’s effects on the nervous system.

Children of alcoholics (COA’s) are at higher risk than the general population for developing alcoholism.^1^ Evidence suggests that this risk is influenced by genetic factors (Hesselbrock 1995). Researchers have identified several biological traits that appear to be genetically transmitted along with vulnerability to alcoholism. These traits can serve as markers to identify persons at risk and can provide valuable information on the development of alcohol use disorders. Because the processes of addiction occur largely in the brain, many studies have investigated various measures of brain function. Much of this research has focused on the brain’s electrical activity.

Brain function can be assessed using event-related potentials (ERP’s), a special application of the electroencephalogram (EEG). The subject in an ERP study wears a cap in which electrodes are embedded. These electrodes record the electrical activity of the brain at intervals of a thousandth of a second (i.e., in milliseconds [ms]) as the subject responds to specific stimuli (e.g., lights or sounds). Although these recordings are made at the scalp, they record overlapping electrical activity emanating from various brain circuits along pathways from sensory reception to higher cognitive processes. The electrical signals are processed electronically and displayed as wavelike lines with a series of peaks (i.e., positive components) and valleys (negative components). Components are described in terms of their wave height (i.e., amplitude, measured in micro-volts) and the time of their occurrence following presentation of the stimulus (i.e., latency, measured in ms).

## Responding to Significant Stimuli: The P3, or P300, Component

A large positive ERP component (P300, or P3; see figure) that peaks between 300 and 500 ms after stimulus presentation appears to be related to the “significance” of a stimulus. A stimulus can be significant by being relevant to a task (e.g., the subject must press a button whenever the stimulus occurs), by motivational factors (e.g., the subject wins money after responding to the stimulus correctly), or by occurring rarely or unpredictably. Significance is unrelated to the physical features of the stimulus itself (for a review, see Regan 1989).

Studies over the past few decades have found the amplitude of P3 to be significantly lower in abstinent alcoholics than in nonalcoholics (for a review, see Porjesz and Begleiter 1996). This deficit occurs most consistently in association with visual tasks and less consistently with auditory tasks. Researchers originally attributed these low P3 voltages to the cumulative toxic effects of alcohol on the brain. However, evidence increasingly indicates that this is not the case: P3’s remain low in alcoholics even after prolonged abstinence, including among members of Alcoholics Anonymous who have been abstinent for as long as 10 years (Porjesz and Begleiter 1985). Furthermore, low P3 amplitude appears to be related to the number of first-degree^2^ alcoholic relatives in the subject’s family, rather than to the subject’s own drinking history (e.g., quantity and frequency of consumption) (Pfefferbaum et al. 1991). These results suggest that rather than being a *consequence* of years of heavy drinking, low P3 amplitudes predate the development of alcoholism.

**Figure:**
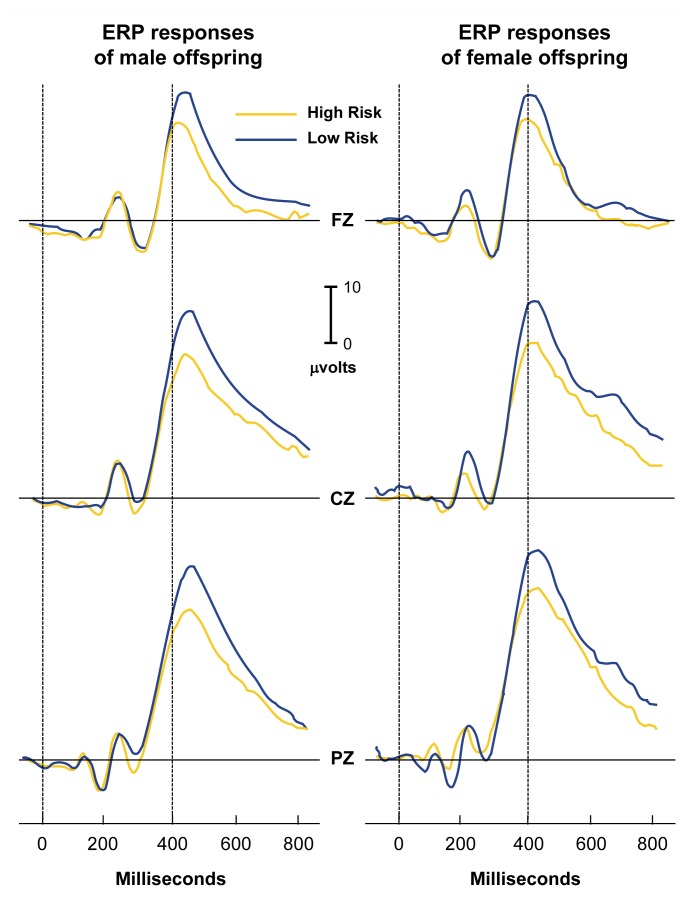
Average event-related potential (ERP) responses (i.e., electrical responses to sensory stimulation of the brain) of male (left) and female (right) offspring of male alcoholics (i.e., high-risk subjects) compared with ERP responses of male and female offspring of nonalcoholics (i.e., low-risk subjects). Electrodes placed against the scalp record ERP’s from specific locations at the front (FZ), center (CZ), and rear (PZ) of the head. The height of the peak, or the amplitude, is measured in terms of the strength of the electrical signal (i.e., microvolts). The P300 component, which is the large positive peak occurring between 300–500 milliseconds after the stimulus, is reduced in voltage in both the male and female offspring of male alcoholics.

## P3 in Nonalcoholic COA’S

The strongest evidence that low P3 amplitudes predate the development of alcoholism comes from ERP studies of subjects at risk for developing alcoholism (see sidebar). Begleiter and colleagues (1984) first reported low P3 amplitudes in sons of alcoholic fathers who were not administered alcohol at the time of the test. This finding has been replicated in several laboratories under many different experimental conditions in both older and younger at-risk subjects. Nevertheless, although most studies have confirmed these results, some studies have not. Low P3’s are most likely to be observed in studies of prepubescent sons of alcoholic men during the performance of difficult visual tasks (Polich et al. 1994). Significantly, however, low P3’s have been detected in all studies that examined offspring of type II alcoholics. Type II alcoholics have an early onset of alcoholism (often during adolescence) with a high rate of relapse and frequently accompanied by petty criminality. This form of alcoholism is thought to have a large genetic component (Cloninger 1987).

**Who Is at Risk?**

Studies of children of alcoholics (COA’s) have produced widely varying results. In part, this variation may reflect the use of inconsistent criteria for determining familial alcoholism. Most investigators refer to a subject as family history positive, or high risk, if the subject has at least one parent who is or was alcoholic. This parent is usually required to be the father, thereby avoiding the problem of potential prenatal alcohol exposure through an alcoholic mother. Some studies, however, define high risk more stringently, requiring the occurrence of alcoholism in several of the subject’s family members or in several generations of the subject’s family. Conversely, other studies require only one symptom of alcoholism in the subject’s father to classify a subject as high risk.

Determining whether a subject’s family member is alcoholic presents additional difficulties, and studies use different criteria for this purpose. Because family members may not be available for diagnosis, researchers must often rely on the subject’s own perceptions or recollections. Consequently, the high-risk group in some studies may include children of “heavy drinkers” or “problem drinkers” in addition to offspring of true alcoholics. This practice may obscure any differences that may exist between the high- and low-risk groups. Similarly, differences in selection criteria for the low-risk (i.e., control) group may also affect study results.

Researchers have applied various strategies to minimize these problems. For example, respondents may be required to identify specific adverse consequences of a family member’s alcohol use, rather than simply noting that the relative has or had a drinking problem. Nevertheless, no single solution exists. Thus, the criteria for subject selection must be considered carefully when evaluating and comparing the results of COA research.

— *Bernice Porjesz and Henri Begleiter*

Interpretation of findings is complicated by conflicting definitions of alcoholism and “at-risk” status across studies. Nevertheless, a recent meta-analysis^3^ of published P3 high-risk studies (Polich et al. 1994) concluded that P3 may have predictive value as an index of vulnerability to alcoholism. Low P3 amplitude in young children has been found to predict alcohol and other drug (AOD) abuse in adolescence (Berman et al. 1993; Hill et al. 1995*a*). Additional confirmation comes from analyses of data obtained by the nationwide Collaborative Study on the Genetics of Alcoholism (COGA). In densely affected alcoholic families (i.e., those in which at least three first-degree relatives are alcoholic), P3 amplitudes were significantly lower in age- and sex-matched offspring of alcoholic fathers than in offspring of alcoholics from randomly selected control families or families with sporadic cases of alcoholism (Porjesz et al. 1996).

Most studies of electrophysiological responses in subjects at risk for alcoholism have focused on males. Results from female offspring have been less consistent (Hill and Steinhauer 1993; Hill et al. 1995*b*). Data from COGA indicate that P3 amplitudes are significantly lower in daughters of alcoholics from densly alcoholic families, although not to the same extent as in sons of alcoholics (SOA’s). These data are illustrated in the figure for both sons and daughters of alcoholic men from densely alcoholic families. Offspring of alcoholic women were excluded from the study to rule out the effects of possible prenatal alcohol exposure.

Although not all COA’s exhibit low P3 amplitudes, a larger percentage of COA’s exhibit low P3’s compared with the rest of the population. COA’s from densely alcoholic families are more likely to exhibit low P3 amplitudes than are other COA’s (Porjesz et al. 1996). Furthermore, subjects with family histories of both alcoholism and antisocial personality disorder (ASPD) have reduced P3 amplitudes. ASPD is characterized by a pattern of irresponsible and antisocial behavior, such as physical fighting or stealing, that begins in childhood or early adolescence and continues into adulthood. Both family history of alcoholism and ASPD are factors that increase the risk of developing alcoholism (Hesselbrock et al. 1993). Thus, the low P3 amplitude seems to be a robust finding that characterizes persons at risk for alcoholism and may provide a vulnerability marker for alcoholism.

## P3 in COA’S in Response to Alcohol

Several studies have examined the effect of alcohol consumption on P3 amplitude (for a review, see Porjesz and Begleiter 1996). In one study (Porjesz and Begleiter 1996), P3 responses were measured in SOA’s and in men who had no first- or second-degree^4^ alcoholic relatives. Drinking histories did not differ between groups. Before testing, the subjects consumed sufficient alcohol to raise their blood alcohol concentration (BAC) to approximately 0.06 to 0.07 percent, which is close to the legal limit for intoxication in most States (0.08 percent). Sons of alcoholics exhibited a larger percentage decrease in P3 amplitude than did sons of nonalcoholics during the ascending phase of the BAC (i.e., when blood alcohol levels are climbing). This pattern may indicate greater sensitivity in the high-risk group during the ascending phase of the BAC, a hypothesis proposed by Newlin and Thomson (1990). Alcohol consumption prolonged the latency of the P3 component in both groups. However, during the descending phase of the BAC (i.e., when blood alcohol levels are dropping), the P3 latencies of the high-risk subjects recovered to prealcohol levels more rapidly than did those of the low-risk subjects (see also Schuckit et al. 1988).

## Implications of Low P3 Amplitude in COA’S

Evidence suggests that the reduced amplitude of P3 in high-risk subjects may serve as a marker of susceptibility to alcoholism. One required characteristic of a marker is heritability; analysis of data from COGA suggests that P3 amplitude may be genetically influenced. Researchers are continuing to study COA’s from densely affected families who participated in COGA to confirm whether COA’s who exhibit low P3 amplitudes are most at risk for developing alcoholism.

In addition to serving as a marker, ERP data may elucidate mechanisms of alcohol’s effects on the nervous system. For example, COA’s tend to respond with low P3 amplitudes to both significant and nonsignificant stimuli. This undifferentiated mode of responding reflects an inefficiency in brain processing. In healthy people, the brain constructs a memory template of nonsignificant stimuli against which to compare each new stimulus. COA’s at risk, however, evaluate each incoming stimulus anew.

Evidence from animal studies indicates decreased nerve cell activity in response to repeated stimuli, suggesting selective inhibition of nerve cells responding to familiar information (Miller et al. 1991) and increased efficiency of communication among nerve cells responding to novel stimuli. The more probable a stimulus, the smaller the P3, whereas the more subjectively improbable a stimulus, the larger the P3. COA’s at risk do not manifest this selective nerve cell inhibition.

One is tempted to speculate about the relationship between this underlying neurophysiological disinhibition and the behavioral disinhibition commonly observed in alcoholics and their offspring. Studies following subjects over a period of time (i.e., longitudinal studies) consistently identify a cluster of behavioral traits that significantly predict high levels of alcohol consumption or abuse. These traits are described as disinhibited, undercontrolled, impulsive, and aggressive (Cloninger et al. 1988). Both ASPD and family history of alcoholism predispose people to alcoholism, and persons in both groups manifest low P3’s (Hesselbrock et al. 1993). The genetic predisposition for alcoholism may be caused by an increase in brain hyperexcitablity in COA’s.

Vulnerability markers are not necessarily specific for alcoholism, and not all persons who exhibit these markers will abuse alcohol. However, evidence suggests that people at risk for developing alcoholism can be distinguished from those not at risk by electrophysiological measures. Because these measures may be genetically determined, a predisposition or vulnerability to alcoholism is probably inherited. The interaction of genetic vulnerability with environmental factors determines whether a person develops alcoholism.

## Footnotes

1 Throughout this article, terms such as “alcohol abuse,” “alcoholism,” and “heavy drinking” are used. Because of the overlap among these terms in the alcoholism literature, the wording in each case is based on the terms used in the reference cited.

2 First-degree relatives include parents, children, and siblings.

3 A meta-analysis is a statistical evaluation of data compiled from several studies.

4 Second-degree relatives include grandparents, aunts, and uncles.
